# Up-regulation of heme oxygenase-1 after infarct initiation reduces mortality, infarct size and left ventricular remodeling: experimental evidence and proof of concept

**DOI:** 10.1186/1479-5876-12-89

**Published:** 2014-04-05

**Authors:** Claudia Kusmic, Cristina Barsanti, Marco Matteucci, Nicoletta Vesentini, Gualtiero Pelosi, Nader G Abraham, Antonio L’Abbate

**Affiliations:** 1 CNR Institute of Clinical Physiology, Via G Moruzzi 1, 56124 Pisa, Italy; 2 Institute of Life Sciences, Scuola Superiore Sant’Anna, Pisa, Italy; 3 Marshall University School of Medicine, Huntington, WV, USA

**Keywords:** Myocardial infarction, Coronary microvascular reactivity, Left ventricular vascularity, Ventricular remodeling, Connexin-43, Cobalt protoporphyrin IX, Tin mesoporphyrin

## Abstract

**Background:**

Up-regulation of HO-1 by genetic manipulation or pharmacological pre-treatment has been reported to provide benefits in several animal models of myocardial infarction (MI). However, its efficacy following MI initiation (as in clinical reality) remains to be tested. Therefore, this study investigated whether HO-1 over-expression, by cobalt protoporphyrin (CoPP) administered after LAD ligation, is still able to improve functional and structural changes in left ventricle (LV) in a rat model of 4-week MI.

**Methods:**

A total of 144 adult male Wistar rats were subjected to either left anterior coronary artery ligation or sham-operation. The effect of CoPP treatment (5 mg/kg i.p. at the end of the surgical session and, then, once a week for 4 weeks) was evaluated on the basis of survival, electro- and echocardiography, plasma levels of B-type natriuretic peptide (BNP), endothelin-1 and prostaglandin E2, coronary microvascular reactivity*,* MI size, LV wall thickness and vascularity. Besides, the expression of HO-1 and connexin-43 in different LV territories was assessed by western blot analysis and immunohistochemistry, respectively.

**Results:**

CoPP induced an increased expression of HO-1 protein with >16 h delay. CoPP treatment significantly reduced mortality, MI size, BNP concentration, ECG alterations, LV dysfunction, microvascular constriction, capillary rarefaction and restored connexin-43 expression as compared to untreated MI. These functional and structural changes were paralleled by increased HO-1 expression in all LV territories. HO activity inhibition by tin-mesoporphyrin abolished the differences between CoPP-treated and untreated MI animals.

**Conclusions:**

This is the first report demonstrating the putative role of pharmacological induction of HO-1 following coronary occlusion to benefit infarcted and remote territories, leading to better cardiac function in a 4-week MI outcome.

## Background

Myocardial infarction (MI) is a life-threatening dynamic process initiating with coronary occlusion and frequently progressing towards chronic heart failure in survivors. In recent years much research has been devoted to revascularization of ischemic territory by both pharmacological and mechanical interventions in the very acute phase, in order to minimize ischemic necrosis and infarct size and reduce early mortality. However, beyond the initial phase, the final outcome of MI is conditioned by a long series of biological processes, which on one hand modulates loss and regeneration of myocardial tissue in the infarcted territory, and on the other remodels the remote, viable myocardium with a relevant progressive loss of function and failure. Noticeably, the unfavorable outcome of MI is primarily related to the activation of the neuro-humoral system but only partially to infarct size. The well-documented beneficial effects of long-term treatment with beta-blockers, diuretics, ace-inhibitors and angiotensin receptor blockers support this view [1-3]. Moreover, among biological factors intervening in structural and functional modifications that characterize the infarcted heart, inflammation and oxidation play a prominent role during the entire time course of MI, from early necrosis to heart failure [4-7]. In this study we have focused on the potential use of pharmacological intervention too late to save irreversibly injured ischemic myocardium, but timed to limit additional myocardial loss due to inflammatory, oxidative and apoptotic processes [8,9], accelerate the repair process and reduce ventricular remodeling.

The heme oxygenase-1 (HO-1) gene has cytoprotective properties mediated by its anti-oxidative, anti-inflammatory and anti-apoptotic effects. In animal models of MI, HO-1 over-expression in either transgenic [10-12] or transfected rodents [13-15] or even pre-treatment with cobalt protoporphyrin IX (CoPP) [16], a powerful and widely used inducer of HO-1 expression [17,18], reduced infarct size as well as ventricular remodeling, enhanced endothelial function, promoted neoangiogenesis and restored cardiac metabolism. Thus, it is well established that HO-1 over-expression, when present at the time of coronary occlusion, is able to minimize myocardial damage and improve outcome through a series of cytoprotective activities and reparative processes involving formation of new vascular structures and myocytes [16]. Unfortunately, the above experimental conditions are far from the clinical reality, which allows treatment only *after* infarct initiation. To the best of our knowledge the only documentation of the beneficial effect of post-MI HO-1 over-expression comes from Lin and coworkers [19] who showed that invasive injection of recombinant AAV bearing HO-1 gene into the border-zone early after induction of MI in mice, promoted neovascularization in the ischemic region and significantly limited left ventricular (LV) fibrosis and dysfunction at 4 weeks.

Thus, in view of a potential novel pharmacological challenge, the aim of our study was to assess whether administration of a pharmacological HO-1 activation, when chances to save ischemic myocardium are greatly reduced or even nil, has any effect on MI outcome. This is a ‘proof of concept’ experimental controlled trial (treated vs untreated vs sham MI). We hypothesized that when ischemic damage was already irreversible, HO-1 over-expression would be able, by its well-documented anti-oxidant, anti-inflammatory, anti-apoptotic and angiogenetic effects, to positively modulate those post-ischemic phenomena that (beyond the initial ischemic damage) significantly contribute to final infarct size, left ventricular dilation and remodeling, and progression towards heart failure. In order to induce HO-1 over-expression we administered CoPP at the end of the surgical procedure of LAD ligation in the rat, following the preliminary documentation that CoPP up-regulates HO-1 protein expression with a delay longer than 16 h.

## Methods

### Ethics statement

Animals used in this investigation conformed to the recommendations in the *Guide for the Care and Use of Laboratory Animals* published by the US National Institutes of Health (NIH Publication No. 85–23, revised 1996) and the protocol was approved by the Animal Care Committee of the Italian Ministry of Health (Endorsement n.135/2008-B). All surgery was performed under anesthesia, and all efforts were made to minimize suffering.

### Animals

Male Wistar rats were either bred in our local animal husbandry facility or purchased from Harlan Italy s.r.l (Udine, Italy). Animals were housed in an environment with controlled 12 h/12 h light/dark cycle, temperature (21 ± 0.5°C) and relative humidity (55% ± 2%) and fed with 4 RF 18 standard rat diet for long-term maintenance (Mucedola, Milano, Italy). Water was available ad libitum.

### Cobalt protoporphyrin administration

Cobalt protoporphyrin (CoPP, Frontier Scientific Inc., Logan, UT, USA), a well-known inducer of HO-1 expression [17,18] was administered via intra-peritoneal injection. We prepared a 5 mg/ml CoPP stock solution in TRIS buffer (pH 8.0); handling of CoPP solution was performed in the dark due to its sensitivity to light. The injected dose was 5 mg/kg body weight.

In order to preliminarily assess the time course of HO-1 protein synthesis after a single injection of CoPP (5 mg/kg), we monitored cardiac expression of HO-1 protein by Western blot analysis in 10 control rats sacrificed at different times (0, 8, 16, 24 and 48 h) following CoPP administration (n = 2 for each time point) (see details in Additional file 1: S1). The expression of HO-1 protein had increased by 16 h and continued to increase through 48 h (see Additional file 1: Figure S1A). In addition, to exclude that the rise of HO activity in the heart forerun the increase in HO-1 abundance (16 h), we also measured the HO activity in myocardial tissue (Additional file 1: Figure S1B) at 0 and 8 hours following CoPP administration (n = 3 for each time point). No increase in HO activity was observed at 8 h.

### Tin mesoporphyrin administration

Tin mesoporphyrin IX (SnMP, Rockfeller University, New York, NY, USA), an inducer of HO protein synthesis [20], is considered a potent inhibitor of the activity of both preformed and newly synthesized enzyme [21,22]. SnMP was administered via intra-peritoneal injection at a dose of 8 mg/kg body weight.

### Induction of myocardial infarction

A total of 144 male Wistar rats 10–12 weeks old and weighing 310 ± 3 g were used in the study. After thoracotomy, MI was induced by ligation of the left anterior descending coronary artery (LAD) (see details in Additional file 1: S2). Immediately after chest closure rats received an intra-peritoneal injection of either vehicle alone (TRIS buffer, MI group, n = 63) or cobalt protoporphyrin alone (CoPP-treated MI group, n = 40) or CoPP in association with SnMP (CoPP + SnMP group, n = 8). Due to the > 16 h delay of CoPP induced HO-1 up-regulation, the adopted scheme of treatment mimicked the clinical condition of a pharmacological intervention well after initiation of MI. Thereafter, in order to sustain HO-1 over-expression, additional doses of CoPP or vehicle were administered i.p. once a week over a 4-week study period. Sham-operated rats underwent all surgical procedures except LAD ligation and received the same intra-peritoneal treatment with vehicle (Sham group, n = 30). In the CoPP + SnMP group, SnMP was administered i.p. every second day [23].

The number and times of spontaneous deaths during the 4 weeks were carefully recorded. Out of 144 animals, three died during surgery due to irreversible ventricular fibrillation and were not allocated to any group. Thus the remaining 141 are included in the study. At 4 weeks, a total of 117 rats survived and entered the morpho-functional study. Allocation of animals to different groups and procedures is detailed in the Additional file 1: S3 and Additional file 1: Table S1.

### Electrocardiographic study

ECG was recorded at 2 kHz sampling rate and heart rate was calculated using the Power Lab monitoring system (ML135 PowerLab/8SP) equipped with ML135 Dual Bio Amp and MLA0112 ECG Lead Switch Box (ADI Instruments Ltd., Oxford, UK). ECG recordings were continuously acquired before (basal condition) and during surgery up to 60 min. Arrhythmias were classified according to the Lambeth Conventions [24] and their severity was scored (range 0–5) [25] as detailed in the Additional file 1: S4. Moreover, an extensive ECG analysis was carried out at 4 weeks after infarction as previously described [26]. The ECG parameters studied were: heart rate, frontal QRS axis (Â_QRS_) using D1 and AVF leads, and QRS amplitude index (I_QRS_), i.e., the sum of positive or negative peak deflections of the QRS complexes in D1, D2 and D3, and QRS duration (T_QRS_). ECG at 4 weeks was compared to the basal one.

### Echocardiographic study

Echocardiographic studies were performed 4 weeks after infarction with a portable ultrasound system (MyLab 25, Esaote SpA, Genova, Italy) equipped with a high frequency linear transducer (LA523, 12.5 MHz). Under intra-peritoneal anesthesia, as previously described, images were obtained from the left parasternal view. A short-axis 2-dimensional view of the left ventricle (LV) was taken at the level of papillary muscles to obtain M-mode recording. Anterior (infarcted) and posterior (viable) end-diastolic and end-systolic wall thicknesses, systolic wall thickening, and LV internal dimensions were measured following the American Society of Echocardiography guidelines. Parameters were the mean of measurements of three consecutive cardiac cycles. The global LV systolic function was expressed as fractional shortening (FS%).

### Plasma determination of BNP, ET-1, and PGE2

Plasma samples were assayed to determine the circulating levels of B-type natriuretic peptide (BNP), endothelin-1 [27,28] and prostaglandin E2 [29], as detailed in the Additional file 1: S5.

### Ex vivo assessment of microvascular reactivity

Microvascular coronary resistance (CR) was evaluated 4 weeks after surgery in the isolated beating heart in Langendorff configuration. This procedure has been previously described in detail [30].

Briefly, two side-arms in the perfusion line, located close to the heart inlet, allowed switching between two reservoirs set at normal (70 mmHg) and low (30 mmHg) pressure. Coronary flow was continuously measured with a flowmeter (model T106, Transonic System Inc, Ithaca, NY, USA) and by measurement of effluent volume with a calibrated pipette. Coronary resistance was calculated as input pressure divided by coronary flow per gram of myocardial tissue (mmHg*g*min/ml). As in infarcted hearts the injection of Evans blue dye into the aortic root did not color the infarcted tissue at macroscopic morphometry (in the Additional file 1: Figure S2), the weight of perfused myocardium was calculated as the total ventricle weight minus the necrotic myocardium.

After an initial 15-min period of stabilization, not included in the analysis, CR was measured in two alternative protocols, (i) at the onset and at the end of 65 min of 70 mmHg perfusion pressure (ii) at 20 min of low perfusion pressure (30 mmHg) and early after reperfusion at 70 mmHg (peak hyperemia).

### Tissue harvesting and macroscopic morphometry

Four weeks after surgery, under deep anesthesia, hearts were arrested in diastole by a lethal KCl injection, and left ventricles were weighted and cut in transversal and parallel slices about 2 mm thick. To enhance the contrast between viable and infarcted myocardium, fresh slices were incubated with triphenyltetrazolium chloride 1% solution at 37° for 10 min. Slivers were photographed with a digital camera and the images processed by dedicated image software (MIAO, Myocardial Infarcted Area Outline), which measured the value of the infarcted area in radiants encompassing the pale area and expressed it as a percentage of the entire left ventricle (360°). The thickness of the central infarcted area and of its opposite wall were measured in each animal (see details in Additional file 1: S6).

Next, in each slice, cardiac tissue was divided into four distinct areas, which were frozen and analyzed separately: a) right ventricle wall; b) left ventricle posterior wall, opposite to LAD territory (remote zone); c) border region to LAD area (border zone); d) central zone of the infarcted area.

In addition, the abdomen was incised and four samples from the right and median lobes of the liver were excised and flash-frozen to determine tissue HO activity.

### HO activity measurement in the liver

To define the efficacy of CoPP in inducing HO-1 expression, HO activity was determined in rat liver microsomes of each MI group by measuring bilirubin as previously described [31]. Details of the procedure are reported in the Additional file 1: S7.

### HO-1 expression

Heart tissue from different cardiac regions was separately homogenized and blotted on PVDF membrane as detailed in the Additional file 1: S8. The membranes were probed with rabbit anti-HO-1. Rabbit anti β-actin or rabbit anti GAPDH antibodies were used to probe the reference proteins. After incubation with HRP-conjugate secondary antibody enhanced chemiluminescence detection was performed and digital images were acquired for densitometry analysis of the bands by using “open source” program Image J (National Institute of Health, Bethesda MD, USA). Protein-specific bands were normalized to the protein loading staining β-actin or GAPDH.

### Apoptosis

Possible effect of CoPP on cardiac apoptosis was explored in an especially dedicated additional set of animals (n = 18, 6 sham, 6 untreated MI and 6 CoPP-treated MI rats). Rats were sacrificed at 16 and 24 h, times proved to correspond to the onset-time of HO-1 over-expression (n = 3 for each time in each group). Myocardial tissue homogenates from infarcted, border and remote regions were processed to assay caspase 3 activity (Caspase 3 Colorimetric Assay Kit, AbCam, Cambridge, UK) and to measure BCL-2 levels (custom made rat BCL-W ELISA kit, RayBiotech, Inc, Georgia, USA).

### Immunohistochemical analysis (connexin 43 and vascularity)

Hearts were arrested in diastole at 4 weeks, ventricles were weighted and fixed in 10% buffered formalin (see details in the Additional file 1: S9). Cardiomyocyte junctions were identified by immunoperoxidase staining, using a polyclonal rabbit anti-connexin 43 (Cx43) primary antibody (AbCam, Cambridge, UK); arterioles and capillaries were labeled with anti-smooth muscle actin (α-SMA) primary antibody (SantaCruz, Dallas, USA) or anti-PECAM 1 (CD31) primary antibody (SantaCruz, Dallas, USA) respectively. Sections were counter-stained with hematoxylin and examined using light microscopy (Olympus BX43) at 10× to 40× original magnification and digitized by a video system (Olympus DP20 camera) interfaced to a computer with dedicated software (CellSens Dimension, Olympus) for image acquisition and morphometric and/or color analysis. Cx43 was quantified as the percent of myocardial area occupied by positive staining (calculated by averaging the values from ten fields at 10× magnification) for each LV area. The density (number/mm^2^) of arterioles (10–100 μm in diameter) was counted by averaging values from eight fields randomly chosen for each LV area at 20× magnification. The same was done for capillaries density (<10 μm in diameter), except magnification 40×.

### Statistical analysis

All experimental values are expressed as the mean ± standard error of the mean (SEM). On the basis of the Kolmogorov-Smirnov test (p > 0.05), the visual inspection of their histograms, and the normal Q-Q plots we assumed that our data were approximately normally distributed.

Individual means of different groups were compared by one-way or two-way analysis of variance (ANOVA) followed by *post hoc* Newman-Keuls tests. To take uneven variances into account, for comparisons between groups of unequal numbers, Welch’s correction for the Student’s t-test was used. Correlation analysis was performed using Spearman’s test. Paired or unpaired t-test and chi-square test were used as appropriate. A value of p < 0.05 was considered statistically significant.

All analyses were done with the statistical computer program StatView 5.0 (Abacus Concepts, Berkeley, CA, U.S.A.).

## Results

### Mortality

Among the 141 animals who survived the surgical procedure, overall mortality at 4 weeks was 3% in the sham group, 25% in the vehicle MI group, 12% in the CoPP-MI group and 25% in the CoPP + SnPP-MI group (in the Additional file 1: Table S1), with vehicle-MI significantly different from CoPP-treated MI rats (χ^2^ = 3.89, p = 0.048) but not different from CoPP + SnMP MI group (χ^2^ = 0.167, p = 0.6831). A comparison of vehicle vs CoPP MI mortality at 24 h (early) and at 72 h (intermediate mortality) showed that cumulative mortality was similar in the two groups at 24 h (6 vs 5%) but had diverged at 72 h (19 vs 7% respectively, χ^2^ = 6.67, p = 0.0098) supporting the assumption of a ‘late’ biological effect of our pharmacological intervention.

### Electrocardiography

In MI groups severe ventricular arrhythmias occurred 5 to 20 min after coronary occlusion. The average severity score was no different in vehicle when compared to CoPP and CoPP + SnMP MI groups (3.35 ± 0.15, 3.35 ± 0.11, and 3.33 ± 0.10 respectively) suggesting the absence of an apparent anti-arrhythmogenic effect of CoPP in the first 20 min of MI. At 4 weeks, only sporadic arrhythmias were observed, never exceeding a score of 0 in all groups.

QRS morphology, 4 weeks after surgery, showed a difference between sham and vehicle MI animals with a rightward shift (> 90°) of the frontal QRS axis, reduction in QRS amplitude index, and prolongation of QRS interval (Table 1). CoPP treatment significantly decreased the rightward shift with 53% of cases of Â_QRS_ axis < 90°, preserved QRS amplitude index, partially restored QRS duration. Concurrent SnMP administration cancelled out the CoPP effect and abolished ECG morphological differences relative to the vehicle MI group.

**Table 1:** In vivo heart functional parameters of rats by ECG at 4 weeks

	**Sham group**	**MI group**	**CoPP-MI group**	**CoPP + SnMP -MI group**
	**n = 20**	**n = 35**	**n = 25**	**n = 6**
Â_QRS_ (cases >90°)	59° ± 2 (0/20)	128° ± 2^**#^ (35/35)	94° ± 5^**^ (12/25)	125° ± 7^**#^ (6/6)
I_QRS_ (mV)	4.1 ± 0.2	2.6 ± 0.1^**#^	3.6 ± 0.2	2.4 ± 0.2^**#^
T_QRS_ (ms)	14 ± 0.6	19 ± 0.5^**#^	15 ± 0.5	18 ± 1.6^**#^

Values are mean ± SEM; n, number of animals tested; Â_QRS_, frontal QRS axis; I_QRS_, QRS amplitude index; T_QRS_, QRS duration; **p < 0.001 vs sham-operated; #p < 0.001 vs. CoPP-treated infarct.

### Echocardiography

The main echocardiographic parameters are shown in Table 2. LV geometry changed in vehicle MI as the result of a progressive LV remodeling over the 4-week study period post coronary artery ligation. Both LV end-diastolic and end-systolic diameters, as well as HR increased in untreated MI compared to sham-operated animals. Moreover, anterior (infarcted) LV wall thickness was reduced in untreated MI hearts, while the posterior (viable) wall thickness was higher than sham rats and CoPP-MI. In vehicle MI the percentage of systolic thickening (an index of contractility) of the anterior and the posterior walls as well as LV fractional shortening was reduced compared to sham animals. CoPP treatment significantly reduced both end-diastolic and end-systolic diameters of infarcted hearts, blunted both the increase in heart rate and the thinning of the anterior wall and improved systolic thickening of both anterior and posterior walls as well as the fractional shortening compared to untreated MI. Concurrent SnMP administration reversed the CoPP effect, abolishing both the LV geometry and functional changes relative to vehicle MI group (Table 2).

**Table 2:** In vivo heart functional parameters of rats by echocardiography at 4 weeks

**Heart function**	**Sham group**	**MI group**	**CoPP-MI group**	**CoPP + SnMP MI group**
	**n = 20**	**n = 35**	**n = 25**	**n = 6**
LVEDd (mm)	5.5 ± 0.2	7.4 ± 0.4^**#^	6.3 ± 0.3^*^	8.5 ± 0.4^**#^
LVESd (mm)	2.0 ± 0.2	5.0 ± 0.3^**#^	2.9 ± 0.1	6.0 ± 0.4^**#^
EDAW (mm)	1.6 ± 0.1	1.1 ± 0.1^**#^	1.5 ± 0.1	1.1 ± 0.1^**#^
EDPW (mm)	1.6 ± 0.1	1.9 ± 0.1^**#^	1.6 ± 0.1	1.9 ± 0.7^**#^
SAWT%	71 ± 3	35 ± 2^**#^	60 ± 3^*^	38 ± 5^**#^
SPWT%	70 ± 2	41 ± 2^**#^	69 ± 2	52 ± 4^**#^
FS%	65 ± 3	34 ± 2^**#^	54 ± 2^*^	29 ± 3^**#^
HR	456 ± 7	495 ± 5^**#^	443 ± 6	470 ± 9^#^

Values are mean ± SEM; n, number of animals tested; LVEDd, left ventricular end-diastole diameter; LVESd, left ventricular end-systole diameter; EDAW, end-diastole anterior wall thickness; EDPW, end-diastole posterior (remote) wall thickness; SAWT%, percent systolic anterior wall thickening; SPWT%, percent systolic posterior (remote) wall thickening; FS%. percent fractional shortening. HR, heart rate. *p < 0.05 and **p < 0.001 vs sham-operated; #p < 0.001 vs CoPP-treated infarct.

### Plasma levels of BNP, ET-1 and PGE2

Plasma BNP, ET-1 and PGE_2_ concentrations before treatment (basal value) were 18 ± 5 pg/mL, 19 ± 0.5 pg/mL, and 8.1 ± 0.5 ng/mL, respectively. Changes in plasma concentrations of the three markers 4 weeks after surgery in sham-operated as well as in vehicle- or CoPP-treated infarcted rats are shown in Additional file 1: Table S2. Briefly, MI group exhibited an increase in both BNP (p = 0.06) and ET-1 (p < 0.01) plasma concentration levels. CoPP treatment dampened the post-infarct rise of both parameters to levels akin to those of sham-operated animals. Moreover CoPP caused a significant increase in PGE_2_ circulating levels in comparison to both sham and MI animals.

### Effect of CoPP treatment on microvascular coronary resistance in isolated hearts

*Ex vivo* measurements of CR are shown in Table 3. Hearts from sham-operated rats perfused at constant pressure exhibited only a small increase in CR detectable over the 65-min study period (p < 0.05). In infarcted animals, coronary resistance values were significantly higher than those in the sham group (p < 0.001 vs sham-operated). The bolus administration of papaverine (50 μg), abolished the increase in resistance in infarcted rats, proving its active nature and ruling out the intervention of extra-vascular compressive forces.

**Table 3:** Values of coronary resistance in isolated heart in Langendorff configuration

	**Sham group**	**MI group**	**CoPP-MI group**
Perfusion at constant pressure (70 mmHg)			
Baseline	8.8 ± 0.5	13.4 ± 0.5^**^	9.2 ± 0.5^#^
65 min	12.5 ± 1.3	32.7 ± 3.3^**^	17.8 ± 1.8^#^
Perfusion at low pressure (30 mmHg) and reperfusion			
Baseline	9.3 ± 0.6	13.6 ± 0.7^**^	8.8 ± 0.6^#^
At 20 min low pressure	15.0 ± 1.6	26.1 ± 2.0^*^	13.2 ± 1.5^#^
Reperfusion	8.5 ± 0.5	19.3 ± 0.9^**^	11.5 ± 0.8

Values are mean ± SEM. *p < 0.01 and **p < 0.001 vs sham-operated; #p < 0.01 vs MI group.

CoPP treatment blunted the increased coronary resistance observed in infarcted rats (p < 0.01 vs untreated MI) (Table 3). Treated animals did not differ from sham-operated ones. In sham-operated hearts, hypotension caused an increase in coronary resistance (paradoxical vasoconstriction, p < 0.01 vs baseline). The return to normal perfusion pressure caused a fall in resistance in the first minute (hyperemic response) below baseline (p < 0.001 vs baseline).

As shown in Table 3, in untreated MI hearts, hypotension induced a marked rise in resistance (p < 0.01 vs sham and CoPP-MI). The return to normal perfusion pressure caused a fall in resistance to a value higher than baseline (p < 0.05 vs baseline). The values of CR were higher than those of sham-operated animals (p < 0.001 vs sham-operated rats).

CoPP-treated MI rats showed basal CR values similar to those of the sham group and lower than those of untreated infarct group (p < 0.01 vs untreated infarct). During transient low-pressure ischemia, CoPP treatment in MI rats largely abolished the vasoconstrictive response to hypotension. At the peak, CR was not significantly different from that of the sham group. Reperfusion produced a drop in CR to a level that was no different from that of the sham-operated group.

### Macroscopic morphometry and infarct size

Ventricles of MI group had significantly (p < 0.001) higher weights than those of sham-operated animals (Additional file 1: Table S3). CoPP treatment decreased (p < 0.001) the post-infarct gain in ventricle weight.

**Figure 1:**
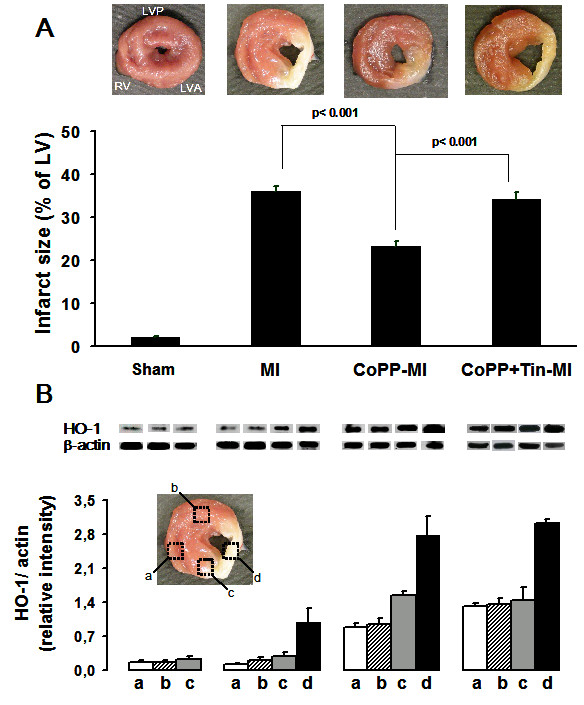
**Histograms of infarct size and HO-1 expression in hearts at 4 weeks after LAD ligation.** From left to right: sham operated (n = 6), vehicle-treated MI (n = 16), CoPP-treated MI (n = 14) and CoPP + SnMP-treated MI rats (n = 6). **A**. Representative freshly-cut transverse sections used for determination of infarct size (upper) and quantitative results on infarct size (lower) expressed as mean ± SE percentage of left ventricle. RV: right ventricle; LVP: left ventricular posterior free wall; LVA: left ventricular anterior free wall. **B**. Representative western blots (upper) and quantitative results from densitometric analysis of HO-1 and β-actin expression (lower) of the corresponding regions shown in the areas outlined by the black boxes and labeled with letters in the representative freshly-cut transverse section: a) right ventricle wall; b) left ventricle posterior wall, opposite to LAD territory; c) border region to LAD area and d) central zone of the infarcted area. Histograms are expressed as ratio between HO-1 and the comparative protein β-actin.

Figure 1A shows MI size evaluated 4 weeks after MI or sham operation. Sham group showed an insignificant area of LV damage (< 2%), mainly due to tissue trauma during sham operation. Four weeks after MI, infarct size was 35.9 ± 1.6% of LV (p < 0.001 vs sham-operated). Thickness of the core infarcted wall was about 43% of the one in the sham group, whereas opposite wall thickness was about 119%, a value significantly (p < 0.05) higher than in sham animals (Additional file 1: Table S3). CoPP treatment significantly reduced MI size to 23.2 ± 1.3% of LV (p < 0.002 vs untreated MI). Moreover, it reduced thinning of the core infarcted region (66% of sham group) and completely prevented the increase in thickness of the opposite region (101% of sham group).

SnMP resulted in MI size of 33.6 ± 1.5% of LV after 4 weeks (p < 0.001 vs CoPP-MI); thickness of the core infarcted wall was 39% while the opposite wall was 120% compared to the sham group.

### Liver HO activity

CoPP increased HO activity in the liver of infarcted rats 2.1-fold as compared to untreated infarcted rats (24.0 ± 5.0 vs 11.6 ± 1.5 pmol bilirubin * min-1 * mg-1, p < 0.05).

SnMP-treated animals exhibited minimal HO activity (0.1 ± 0.08 pmol bilirubin * min-1 * mg-1, p < 0.001 vs both vehicle and CoPP treated-MI groups).

### HO-1 expression in the heart

In sham-operated hearts HO-1 protein levels in different myocardial regions were low (Figure 1B). Conversely, in the untreated MI group 4 weeks after LAD occlusion, HO-1 expression levels were significantly increased in the central zone of the infarcted area. CoPP treatment resulted in an increase of HO-1 protein levels in all regions compared with both sham and untreated MI groups (p < 0.001 vs the other groups). Nonetheless in the CoPP-treated MI group there was a heterogeneous expression of HO-1 protein in the different myocardial regions with the highest levels in the core of the infarcted area (p < 0.05 vs border zone and p < 0.001 vs both LV remote zone and RV). Likewise, HO-1 levels in the border region were about twofold higher than in both remote area and right ventricle (p < 0.05). The relative values of HO-1 expression level in each region of CoPP-treated MI group were: IZ > BZ > RZ = RV.

The addition of SnMP induced a regional pattern and levels of HO-1 expression in each cardiac region akin to those of CoPP alone.

### Connexin-43

**Figure 2:**
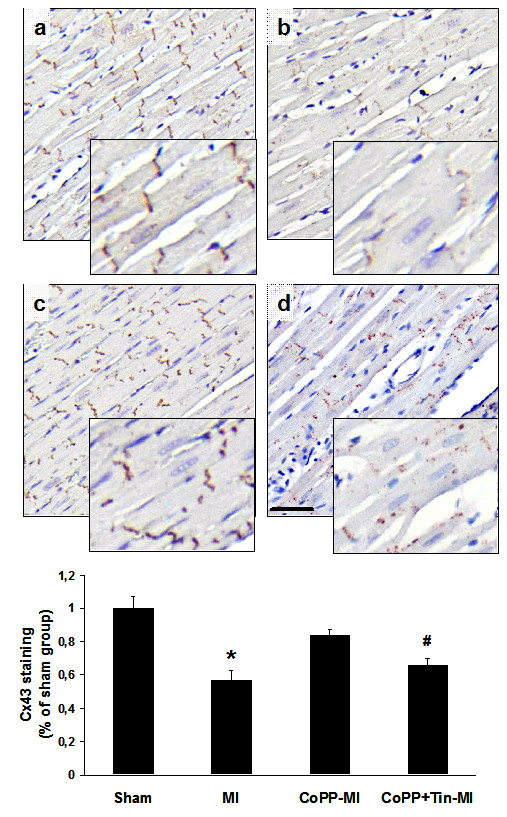
**Distribution of connexins 43 in the remote region at 4 weeks after LAD ligation.** Upper panels: representative immunostaining for Cx43 from sham-operated **(a)**, vehicle treated MI **(b)**, CoPP-treated MI **(c)**, and CoPP + SnMP-treated MI groups **(d)** at low magnification (10×, calibration bar: 40 μm) and at higher magnification (400×, insets). Lower panel: histograms of the proportion of the total cell area occupied by Cx43 immunoreactive signal in the remote region. Data are expressed as means ± SE. *p < 0.001 compared with both sham-operated and CoPP-treated MI groups; #p < 0.02 compared with sham-operated group.

Immunohistochemistry showed a discrete pattern of connexin-43 staining in LV sections of sham-operated rats, predominantly localized to the myocyte-myocyte junction corresponding to intercalated discs. As compared to sham hearts, quantitative analysis of connexin-43 staining showed a significantly decreased Cx43 in untreated MI rats both in the remote region (57% ± 8% of sham, p < 0.001 vs sham group) (Figure 2) and in the border zone (51% ± 10% of sham, p < 0.001 vs sham group) (data not shown). CoPP treatment resulted in a significant conservation of Cx43 expression in the remote region (84% ± 8% of sham, p < 0.001 vs MI vehicle group) and also in the border areas (75% ± 16% of sham, p < 0.02 vs MI vehicle group).

In the SnMP treated group, Cx43 staining was significantly lower in both the remote zone (66% of sham, p < 0.02 vs sham) and in the border zone (63% of sham, p < 0.05 vs sham), similar to untreated MI hearts.

### Arteriolar and capillary density

**Figure 3:**
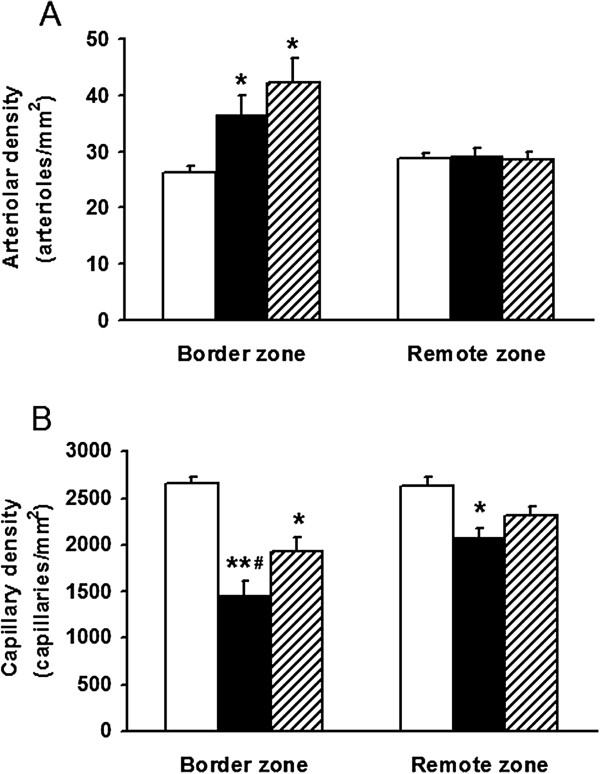
**Histomorphometric analysis of arteriolar and capillary density in the border and the remote region at 4 weeks after LAD ligation.** Histograms of arteriolar density **(A)** and capillary density **(B)** in the border (BZ) and the remote (RZ) zone of sham operated animals (white bar, n = 6), MI group (black bar, n = 6) and CoPP-treated MI group (dashed bar, n = 6). Data are expressed as means ± SE. *p < 0.05 and **p < 0.001 compared with sham operated; #p < 0.05 compared with CoPP-treated MI groups.

Figure 3 shows the results of morphometric analysis in untreated and in CoPP treated animals. In untreated MI, as compared to the corresponding zones in sham hearts, we observed the increase in arteriolar density in the border zone (140%, p = 0.02) as opposite to the capillary rarefaction in the border (55%, p < 0.001) as well as in the remote zone (79%, p < 0.05). CoPP treatment further increased arteriolar density in the border zone (162%, p < 0.05) and corrected capillary rarefaction partially in the border zone (69%, p < 0.05 vs untreated MI) while completely in the remote zone (86% ns).

### Relationship between measured variables in the untreated infarcted animals

Correlation matrix of ECG diagnostic signs, anatomical parameters, indices of LV function, and tissue and humoral biomarkers (Cx43 and BNP respectively) in untreated infarcted animals is shown in Additional file 1: Table S4. Infarct size positively correlated with Â_QRS_, indicating that the rightward shift of the frontal axis is a reliable index of anterior infarct size in the animal model used, and with LV enlargement. Remote wall thickness, a marker of LV remodeling, negatively correlated with LV function and Cx43 and positively with BNP. Functional parameters of LV correlated well with ECG diagnostic signs, with Cx43 staining and BNP.

### Effect of HO-1 over-expression on apoptosis early after MI

Hearts untreated and CoPP-treated rats sacrificed at 16 and 24 h after MI were tested for caspase 3 activity and BCL-W expression level and compared with sham hearts. (n = 3 for each time in each group). Results did not evidence any significant difference between untreated and CoPP-treated MI rats either in the caspase 3 activity or in the level of BCL-W expression. As the BCL-W level is concerned, a significant increased level of BCL-W at both 16 h (p < 0.05) and 24 h (p < 0.01) was found in the infarct zone irrespective of treatment with respect to sham operated animals.

## Discussion

The rat model of MI we adopted in the present study manifested large infarct size but relatively low mortality (36% of LV and 25% respectively at 4 weeks), thus allowing the study of the long-term efficacy of HO-1 activation many hours after MI initiation on both mortality and LV remodeling in survivors. We preferred permanent LAD occlusion to an ischemia-reperfusion model due to the higher consistence of anatomical and functional findings in this model compared to the much higher variability proper to the ischemia-reperfusion one. Moreover, it is a more challenging model for testing HO-1 efficacy on outcome when a definite ischemic necrosis is already established. The priority of our ‘proof of concept’ experimental controlled trial was to assess in evolving myocardial infarction whether late HO-1 over-expression (i.e., at the time when irreversible ischemic damage is already established) is still able to improve outcome (mortality, ventricular dysfunction and left ventricular remodeling). In fact, in absence of such evidence the previously documented beneficial effect of HO-1 over-expression before MI initiation has no practical translational meaning due to the clinical inapplicability of any pharmacological pre-treatment in MI.

In our model, CoPP administration at the end of surgical procedures was the way of over-expressing HO-1 late after MI initiation (> 16 h). The beneficial effect of such treatment on infarct size, cardiac function and ventricular remodeling implies the ‘late’ occurrence of non-obvious cellular phenomena that can be addressed by pharmacological intervention in HO-1 up-regulation and positively modify final outcome. The mechanisms of such effects were not the object of the present study and deserve dedicated investigation. Out of the previously reported beneficial effect of HO-1 over-expression (anti-oxidative, anti-inflammatory and anti-apoptotic effects on one hand and enhanced endothelial function and neoangiogenesis on the other [10-16]) we could document its positive effect on border zone arteriolar density and capillarity in both border and remote zones. Conversely, we were unable to document any effect of HO-1 activation on early (16 and 24 h) cardiac apoptosis. As the time course of apoptosis following acute MI is still unknown, different results at different times cannot be ruled out.

### Untreated infarct

LAD ligation produced a LV antero-lateral transmural scar, which averaged 35.9 ± 1.6% of the LV 4-weeks after occlusion, confirming previous data [16,32-34].

### ECG, LV function and Connexin43

In untreated MI animals the frontal QRS axis (ÂQRS) shifted to the right, QRS amplitude decreased, while QRS duration increased when compared to sham animals. ÂQRS deviation embodies an imbalance in the cardiac electrical field, due to the loss of an electrically active area, towards a direction divergent from infarct location [26,35,36]. In contrast, the decrease in QRS amplitude reflects the reduction in viable myocardium that contributes to electrical potential generation as well as to ventricular function [26]. Finally, the increase in QRS duration reflects a delay in electrical propagation through the viable myocardium being generally attributed to the derangement of the intra-ventricular Purkinje conduction system and thought to be responsible for asynchrony of contraction and thus loss of ventricular systolic function [37]. However, in the interpretation of ECG changes it should be considered that gap junctions (clusters of channels constructed from connexins, mainly Cx43) are a major determinant of the electrical propagation and electro-mechanical coupling [38,39].

In our study Cx43 staining positively correlated with QRS amplitude and negatively with QRS duration. Previous studies have shown decreased QRS amplitude in genetically restricted Cx43 mice [40,41] and Bacharova et al. have attributed to Cx43 reduction the discrepancy between QRS voltage and LV mass in spontaneously hypertensive rats [42]. Finally, at least two studies have shown a significant prolongation of QRS complex in heterozygous deficient Cx43 mice [38,43].

Regarding myocardial infarction, decreased Cx43 expression and disorganization of the gap junctions in the border zone [44] or in the peri-infarct zone [45] or in both [15,46] have been reported. In contrast, information on Cx43 expression and organization in the remote LV region is very scanty. In line with our findings, decreased Cx43 staining in the myocardium far from any scar has been reported in ischemic patients [47] as well as in the remote region of infarcted mouse heart [48]. Unfortunately previous studies did not focus on the relationship of Cx43 with ECG and LV structural and functional changes in MI but rather on the putative arrhythmogenic role of Cx43 [46,49,50].

### Microvascular reactivity

Compared to sham-operated hearts, untreated infarcted hearts had a higher basal resistance that progressively increased during prolonged perfusion, and higher vasoconstrictive response to hypotension. The vasoconstrictive nature of this increase in resistance was confirmed by its reversal by papaverine. The finding of increased basal microvascular resistance is in agreement with the documented hypo-perfusion of the remote viable region in patients with chronic myocardial infarction [51,52]. In previous *ex-vivo* experiments, we found a similar increase in coronary resistance in control hearts following L-NAME [30] suggesting impaired endothelial function and reduced NO bioavailability in the surviving myocardium. However, the mechanism(s) underlying the further increase in resistance during hypotension (paradoxical vasoconstriction) in the remote zone of infarcted heart remains to be elucidated, especially in relation to the observed significant increase of circulating ET-1 in MI as opposed to PGE_2_.

### Effects of post-occlusion CoPP treatment

The preliminary test on the time course of HO-1 over-expression induced by CoPP i.p. injection in a group of normal rats indicated that HO-1 over-expression became apparent 16 h after CoPP administration, and further increased at 24 and at 48 h (Additional file 1: Figure S1). These results augment previous data from Lakkisto et al. showing a 1.5-twofold increase in HO-1 protein at 24 h following a single i.p. injection of CoPP at the same dose we used, which lasted for 1 week [16]. Continuing increase of HO-1 expression at 48 h confirms a previous report suggesting a long-lasting effect of CoPP on HO-1 over-expression [17]. In MI animals we administered CoPP *after* coronary occlusion, and then once a week for 4 weeks. This treatment schedule pursued the idea of inducing HO-1 expression late after LAD occlusion and sustaining its activation for the 4-week study period. A powerful and prolonged increase in HO-1 expression was evident at 4 weeks in all cardiac regions with the highest level in the infarcted area. Compared to untreated MI, CoPP significantly decreased the rate of spontaneous death beyond 24 h, inferring a beneficial effect of treatment at delayed times only. Compared to untreated MI, CoPP reduced ECG alterations at 4 weeks, according to the decreased size and transmurality of the infarct, as well as to the preserved Cx43 architecture in the remote zone. CoPP treatment improved left ventricular function as assessed *in vivo* by reduced LV volumes, increased LV fractional shortening and increased systolic thickening of the viable wall. The above were associated *in vitro* with reduced infarct size, no clear hypertrophy of the remote myocardium, and preservation of the Cx43 staining and its spatial organization in the remote region. SnMP cancelled the beneficial effects of CoPP, abolishing any differences relative to the untreated MI group. This finding focuses on HO activity as very primarily responsible for the improved conditions in the heart after LAD occlusion. CoPP also prevented the increase in heart rate observed in untreated MI. This finding was likely related to the protective effect of CoPP on ventricular function and its limitation of heart failure.

The reduction of infarct size by CoPP post-occlusion administration is of considerable clinical significance. As HO-1 up-regulation conceivably began well beyond the completion of the ischemic necrosis, a process not susceptible to reversibility, one should consider that the infarct size at 4 weeks in untreated animals was the integrated result of ischemic plus non-ischemic myocardial loss taking place late after ischemic necrosis [9]. Moreover, one should consider additional processes moving in the opposite direction, i.e., repairing and regenerative processes limiting final infarct size. Lakkisto and colleagues found that a single injection of CoPP 24 h before LAD ligation was able to increase cell proliferation and tissue repair and decrease the apoptotic loss of cardiomyocytes in the border area during the first few days after MI [16,53]. This very dynamic scenario of loss and replacement of myocardial tissue makes it reasonable to conceive that the increase in HO activity, although delayed, was able to prevent ‘non-ischemic’ tissue loss, and/or to exalt regeneration of new muscular tissue or both.

Although this study does not allow discrimination between these mechanisms, the higher expression of HO-1 in the infarct area and in the border zone relatively to the other territories, still evident at 4 weeks after the initial event, strongly suggests its role in the healing process of MI. The occurrence of reparative processes sustained by the formation of new vascular structures seems to be supported by the finding of increased density of both arterioles and capillaries in the present study following CoPP.

Moreover, CoPP prevented the increase in CR observed *ex vivo* in the isolated heart of untreated MI hearts. The effects of CoPP seen here may be partly due to the end-products of heme degradation, bilirubin and carbon monoxide, a powerful vasodilator. In addition, CoPP administration abolished the progressive increase of resistance during prolonged perfusion, in agreement with our previous observations obtained in animal models with critical shortage in both NO production and bioavailability [54-56].

## Conclusion

Previous studies have shown the beneficial effect of HO-1 over-expression in animal models of myocardial infarction and have widely explored the numerous underlying molecular mechanisms. However, despite their pathophysiological importance, these studies have no clinical impact since HO-1 over-expression was induced either by genetic manipulation or pharmacological *pre*-treatment, two conditions that are far from the clinical scenario that conceives of treatment only during evolving acute infarction. In the present study, we provided experimental evidence that HO-1 over-expression begun *late after* LAD ligation, and continuing afterward in the healing and chronic phase, is still able to reduce mortality, infarct size, left ventricular dysfunction and remodeling. This emphasizes the dynamic nature of the event ‘infarction’ that cannot be confined to the post-occlusion myocardial ischemic damage but progresses ahead in a continuum of biological processes involving the whole heart.

Our findings support the putative role of pharmacological induction of HO-1 in the clinical setting, where medical therapy is always initiated *after* the onset of infarction, in order to obtain benefits in both infarcted and remote territories, leading to better cardiac function and auspiciously to better medium- to long-term outcome. In this perspective, our results support research on novel pharmacological inducers of HO-1 over-expression in humans.

## Competing interests

The authors declare that they have no competing interests.

## Authors’ contributions

CK conceived, designed and performed in vivo ed ex vivo experiments, analyzed the data and performed statistical analyses, drafted the manuscript. CB performed sample collection, enzyme activity assays, western blot and data analyses. MM performed immunohistochemistry and image analyses. NV provided a major contribution in data analyses and in the design of the manuscript coordination and its writing. GP provided a major contribution in image analyses and helped the manuscript coordination. NGA participated in the design and coordination of all studies and helped to draft the manuscript. AL conceived, designed and coordinated all studies and drafted the manuscript. All authors read and approved the final manuscript.

## Supplementary Material

Additional file 1 **Supplemental material with tables and figures on methodological details and specific results.** The file contains the following issues: **S1.** Time course of HO-1 expression and HO activity following CoPP administration. **S2.** Myocardial infarction. **S3.** Allocation of animals to different groups and procedures. **S4.** Arrhythmia severity scoring rank. **S5.** Plasma determination of BNP, ET-1, and PGE2. **S6.** Macroscopic morphometry. **S7.** HO activity measurement in the liver. **S8.** HO-1 expression (western blot). **S9.** Immunohistochemistry (connexin 43 and vascularity). **S10.** Correlation matrix of variables in untreated MI group. Click here for file
