# Supplementary material for: Up-regulation of heme oxygenase-1 after infarct initiation reduces mortality, infarct size and left ventricular remodeling: experimental evidence and proof of concept
Source: J Transl Med. 2014 Apr 5;12:89. doi: 10.1186/1479-5876-12-89 (PMC4022338; doi:10.1186/1479-5876-12-89)
Supplement: Additional file 1 — Supplemental material with tables and figures on methodological details and specific results. The file contains the following issues: S1. Time course of HO-1 expression and HO activity following CoPP administration. S2. Myocardial infarction. S3. Allocation of animals to different groups and procedures. S4. Arrhythmia severity scoring rank. S5. Plasma determination of BNP, ET-1, and PGE2. S6. Macroscopic morphometry. S7. HO activity measurement in the liver. S8. HO-1 expression (western blot). S9. Immunohistochemistry (connexin 43 and vascularity). S10. Correlation matrix of variables in untreated MI group. [file 1479-5876-12-89-S1.pdf]

Supplemental Material to manuscript JTM-6303285310023772

Up-regulation of heme oxygenase-1 after infarct initiation reduces mortality, infarct size and left ventricular remodeling. Experimental evidence and proof of concept

Claudia Kusmic,[1] Cristina Barsanti,[2] Marco Matteucci,[2] Nicoletta Vesentini,[1] Gualtiero Pelosi,[1] Nader G. Abraham,[3] Antonio L'Abbate[1,2]

[1]CNR Institute of Clinical Physiology, Pisa, Italy, [2]Institute of Life Sciences, Scuola Superiore Sant'Anna, Pisa, Italy, [3]Marshall University, School of Medicine, Huntington, West Virginia, USA

Corresponding author:

Claudia Kusmic - CNR Institute of Clinical Physiology

Via G Moruzzi 1 - 56124 Pisa, ITALY

Phone: +39 050 3153306 - E-mail: kusmic@ifc.cnr.it

Table of Contents

S1. Time course of HO-1 expression and HO activity following CoPP administration

S2. Myocardial infarction

S3. Allocation of animals to different groups and procedures

S4. Arrhythmia severity scoring rank

S5. Plasma determination of BNP, ET-1, and PGE2

S6. Macroscopic morphometry

S7. HO activity measurement in the liver

S8. HO-1 expression (western blot)

S9. Immunohistochemistry (connexin 43 and vascularity)

S10. Correlation matrix of variables in untreated MI group

S1. Time course of HO-1 expression and HO activity following CoPP administration

The time course of HO-1 protein synthesis in cardiac tissue after a single injection of CoPP (5mg/kg) was assessed in 10 control rats sacrificed at 5 different times (0, 8, 16, 24 and 48 h) following CoPP administration (n = 2 for each time point). GAPDH was used as an internal control. The expression of HO-1 protein had increased by 16 h and continued to increase through 48 h (Fig. S1A). Concurrently, due to the shortage of myocardial tissue for further analysis, we measured HO activity in the liver in this group of rats. The increase of HO activity paralleled the increase in HO-1 expression (data not shown). Then, to test the possibility that the rise of HO activity in the heart forerun the increase in HO-1 abundance (16h), we also measured the HO activity in myocardial tissue (Fig. S1B) at 0 and 8 hours following CoPP administration (n=3 for each time point). No increase in HO activity was observed at 8h both in the heart and in the liver, thus confirming the parallelism of the two tissues.

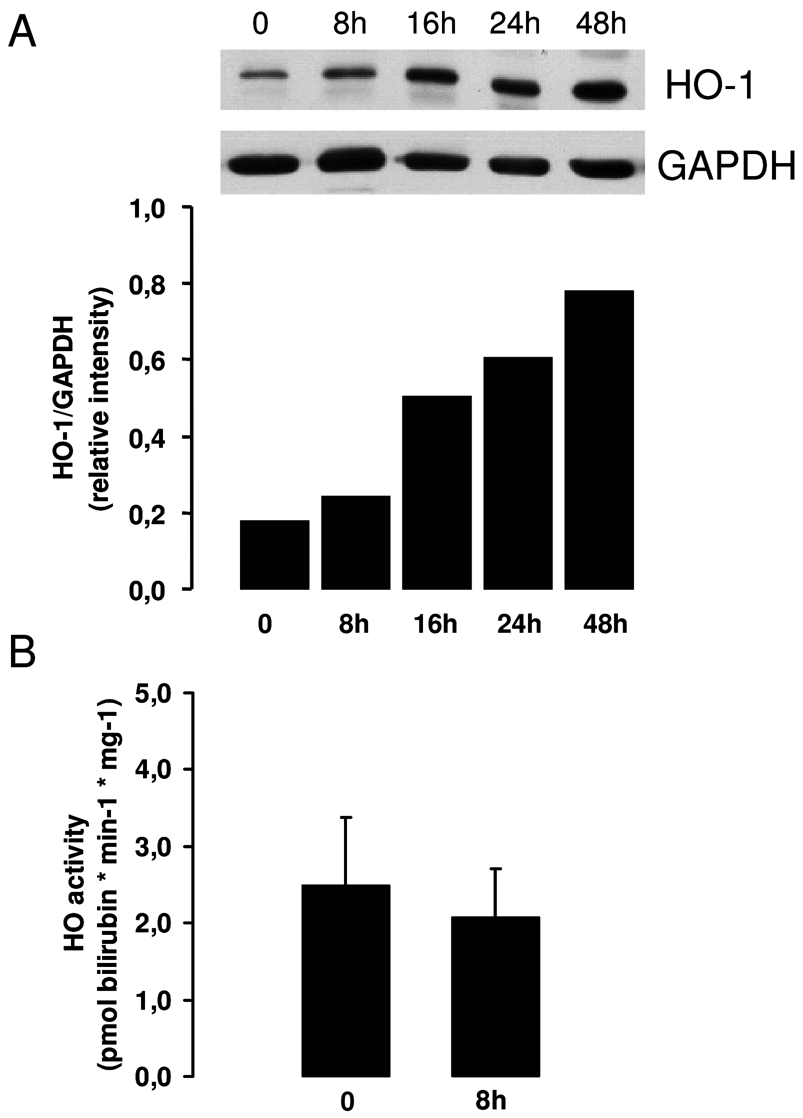

Fig. S1. **Time course of HO-1 protein expression and HO activity in the heart following CoPP administration. A**. Western blots and densitometric analysis in myocardial tissue of 10 control rats sacrificed at 5 different times (0, 8, 16, 24 and 48 h) following a single intraperitoneal injection of CoPP (5mg/kg). Histograms (n = 2 for each time point) are expressed as ratio between HO-1 and the comparative protein GAPDH. **B**. Measurement of myocardial HO activity at times 0 and 8 h documents the lack of HO-1 activation at 8 h following CoPP administration. Data are expressed as the mean ± SE (n=3 in each group).

S2. Myocardial infarction

Rats were anesthetized using Zoletil® + xylazine (50 and 3 mg/kg respectively). A standard limb D1-D3 electrocardiogram (ECG) was monitored with subcutaneous stainless steel electrodes. The rats were connected to a respirator through an oropharyngeal cannula and the heart was rapidly exposed through a left lateral thoracotomy and pericardial incision.

The coronary artery was ligated about 2-3 mm from its origin. Ischemia was confirmed by ST segment elevation and/or regional cardiac cyanosis. The heart was returned to its normal position and the thorax closed.

Post-operatively, all rats were hydrated with physiological saline and given the analgesic buprenorphin 0.05 mg/kg s.c. (Temgesic®, Schering-Plough, Brussels, Belgium) twice a day for 3 days.

After 3 days, all infarcted rats had recovered.

Four weeks after LAD occlusion, the injection of Evans blue dye into the aortic root did not color the infarcted tissue at macroscopic morphometry.

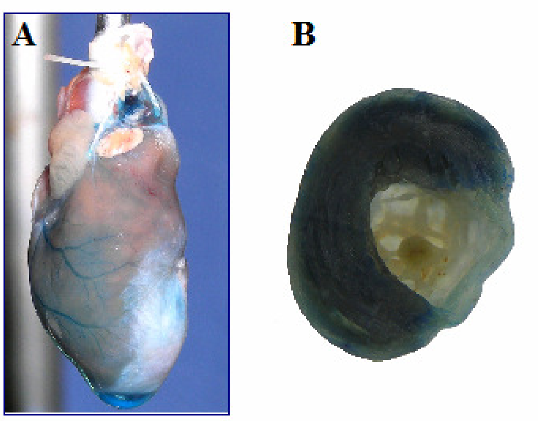

Figure S2. **Isolated infarcted heart injected with Evans blue dye at 4 weeks after LAD ligation.** A: isolated infarcted heart in Langendorff configuration following the injection of the blue dye into the aortic root, viewed from the right ventricle side. The pale scar occupies the mid apical portion of the LV anterior wall. B: freshly cut transversal section of the heart at mid level (4-5 millimeters below the LAD ligation) showing the absence of dye in the scar.

S3. Allocation of animals to different groups and procedures

Table S1 shows the number of animals in each group and their allocation to *in vivo*, *ex vivo*, and *in vitro* protocols. Criteria for allocating animals to different groups and different procedures were: different survival rates in different groups (sham < treated MI < and untreated MI), different intergroup variability (sham < MI), and conflicts between procedures in the same heart (e.g., Langendorff configuration vs morphometry and/or molecular and histochemical analyses). The number of animals in each subset was always sufficient to reach > 90% statistical power. The group of animals (n=18) tested for CoPP effect on cardiac apoptosis at 16 and 24h is not shown.

Table S1. Animal allocation in each experimental group and type of analysis

Survived to surgery	Sham<br><br>n = 30	MI<br>vehicle<br>n = 63	MI<br>CoPP<br>n = 40	MI CoPP<br>+SnMP<br>n = 8	Total<br>141/144
Survival at 4 weeks<br>(% overall mortality)	29<br>(3%)	47<br>(25%)	35<br>(12%)	6<br>(25%)	117
ECG + Echo	20	35	25	6	86
Blood test	20	35	25	n.d.	80
Langendorff,	16	20	12	n.d.	48
Macro-morphometry	6	16	14	6	42
HO-1 expression	4	4	4	4	16
HO-1 activity (liver)	6	6	6	6	24
Histochemistry	6	6	6	6	24

S4. Arrhythmia severity scoring rank

The following scoring system was used to classify the severity of arrhythmias during surgery: 0-50 ventricular ectopic beats (VEBs) score 0; 50-500 VEBs score 1; more than 500 VEBs, or one episode of spontaneously reversible ventricular tachycardia (VT) or ventricular fibrillation (VF) score 2; 2-30 episodes of spontaneously reversible VT and/or VF score 3; more than 30 episodes of spontaneously reversible VT and/or VF score 4; irreversible VF was given a score of 5.

S5. Plasma determination of BNP, ET-1, and PGE2

One ml of blood was collected from the femoral vein under sedation before LAD ligation and before sacrifice 4 weeks later. Plasma samples were assayed to determine the circulating levels of B-type natriuretic peptide (BNP, rat BNP-45 ELISA Kit, (Gentaur GmbH, Aachen, Germany), endothelin-1 and prostaglandin E2 (ET-1 EIA kit and PGE2 EIA kit (Assay Designs Stressgen, Ann Arbor, MI, USA, respectively). We chose to assess these two vasoactive molecules because of the reported negative association between plasma ET-1 levels and cardiac function in heart failure [see ref. 27, 28 of the main text] and because of the recent attribution to cycloxygenase-derived PGE2 of a cardioprotective role in post-infarct remodeling [see ref. 29 of the main text].

Table S2. Plasma concentration of BNP, ET-1 and PGE2 at 4 weeks

	Sham group n = 20	MI group n = 35	CoPP-MI group n = 25
BNP (pg/ml)	21 ± 4	92 ± 12$^{\#}$	36 ± 7
ET-1 (pg/ml)	20 ± 0.6	27 ± 1.5**	23 ± 1
PGE_2 (ng/ml)	8.4 ± 0.3	8.8 ± 0.1$^{\#}$	10.5 ± 0.4*

Values are mean ± SEM; n, number of animals tested. *p < 0.05 and **p<0.01 vs sham-operated; #p < 0.05 vs CoPP-treated infarct

S6. Macroscopic morphometry

After stopping the harts in diastole, left ventricles were weighted and freshly cut in transversal and parallel slices about 2 mm thick. The thickness of the central infarcted area and of its opposite wall were measured in each animal in the (Table S3).

Table S3. Macroscopic morphometric parameters at 4 weeks

	Sham group<br><br>n = 6	MI group<br><br>n = 16	CoPP-MI group<br>n = 14	CoPP+SnMP MI group<br>n = 6
Ventricle weight (g)	1.32 ± 0.3	1.7 ± 0.13$^{**\#}$	1.48 ± 0.13	1.65 ± 0.15$^{**\#}$
WT infarcted (mm)	2.66 ± 0.05	1.15 ± 0.07$^{**\#}$	1.74 ± 0.11*	1.03 ± 0.03$^{**\#}$
WT remote (mm)	2.60 ± 0.05	3.08 ± 0.06$^{**\#}$	2.65 ± 0.06	3.05 ± 0.09$^{**\#}$

Values are mean ± SEM; n, number of animals tested; WT, wall thickness.
*$p < 0.05$ and **$p < 0.001$ vs sham-operated; #$p < 0.001$ vs CoPP-treated infarct

S7. HO activity measurement in the liver

Frozen liver tissue (100 mg) treatment was homogenized on ice and serially centrifuged to separate the microsomal fraction. Samples were aliquoted and stored at -80 °C until assayed. Protein concentration in microsomal fractions was quantitated using the BCA Protein Assay Kit (Pierce, Rockford, IL, USA). HO activity was measured by adding one mg of microsomes to 1 ml of reaction mixture containing 2.5 mM $MgCl_2$, 0.8 mM nicotinamide dinucleotide phosphate, 2 mM glucose-6-phosphate, 20 μM hemin, 3 milligrams of liver cytosol in 100 mM potassium phosphate buffer (pH 7.4) and 1 U/ml glucose-6-phosphate dehydrogenase, incubated at 37 °C for 1 h. The reaction was stopped by the addition of 2 ml ice-cold chloroform and bilirubin was extracted in the chloroform layer. Bilirubin formation was measured

spectrophotometrically (Perkin-Elmer UV/VIS Spectrometer Lambda Bio 40) by calculating the difference in absorbance between 464 nm and 530 nm using an extinction coefficient of 40 $mM^{-1}cm^{-1}$. Readings from samples prepared in the absence of starter enzyme in the reaction mixture served as blanks. HO activity was expressed as picomoles of bilirubin/min per milligram of protein.

S8. HO-1 expression (Western blot)

Heart tissue from different cardiac regions was separately homogenized in a lysis buffer containing (in mmol/l) Tris-HCl 25, NaCl 25, EDTA 1, 0.1 PMSF, pH 7.5. Homogenates were centrifuged at 15,000 x *g* for 10 min at 4˚C. Protein concentration in the supernatant was determined using the BCA protein assay kit (Pierce). Equal amounts of protein (20 µg) were fractionated by 8-10% SDS polyacrylamide gel and blotted onto PVDF (Immun-Blot PVDF Membrane, Biorad). Equal loading was controlled by Ponceau staining. The membranes were probed with rabbit anti-HO-1 (Stressgen, Temecula, CA, USA). Rabbit anti β-actin (Cell Signaling Technology) or rabbit anti GAPDH (Cell Signaling Technology) antibodies were used to probe the reference proteins. After incubation with HRP-conjugate secondary antibody (Cell Signaling Technology), enhanced chemiluminescence detection was performed using the LiteAblot Extend System (EuroClone). Digital images were acquired for densitometry analysis of the bands by using "open source" program Image J (National Institute of Health, Bethesda, MD, USA). Protein-specific bands were normalized to the protein loading staining β-actin or GAPDH.

S9. Immunohistochemistry (Connexin 43 and vascularity)

Ventricles were fixed in 10% buffered formalin for 24-48 h, dehydrated through alcohol series, cleared in xylene and embedded in paraffin. Serial 5- to 7-μm transverse sections starting from the mid-ventricular (1mm distal to the ligature) level were obtained. Tissue slides were pretreated with target retrieval solution (sodium-citrate solution 10 mmol/l, pH 6.0) at 95 °C for 15 min before successive procedures. Endogenous peroxidase was blocked in a solution of 3% H_2O_2 for 15 min at room temperature. After incubation with 5% goat serum for 30 min, sections were incubated with primary antibodies at 37 °C for 1 h followed by 3 washes with PBS. Chromogenic detection of antigens was done by Vectastain ABC system (Vector Laboratories, Burlingame,CA, USA) based on avidin-biotin immunoperoxidase.

S10. Correlation matrix of variables in untreated MI group

In order to explore the relationship among the parameters studied to characterize the MI group from a functional, biohumoral and morphometric point of view, we generated a correlation matrix of variables. R values are reported in Table S4.

Table S4. Correlation matrix (r value) of studied variables in the population of untreated infarcted rats

	$\hat{A}_{QRS}$	I_{QRS}	T_{QRS}	IS	PWT	lvEDd	FS	SPWT	Cx43
I_{QRS}	-0.5 [a]								
T_{QRS}	0.57 [b]	-0.59 [b]							
IS	0.5 [a]	n.s.	n.s.						
PWT	0.68 [a]	-0.59 [a]	0.67 [b]	n.s.					
lvEDd	0.73 [b]	n.s.	n.s.	0.6 [a]	0.7 [b]				
FS	-0.53 [a]	0.69 [b]	-0.5 [a]	n.s.	-0.76 [b]	-0.6 [a]			
SPWT	-0.85 [c]	0.69 [a]	-0.85 [c]	n.s.	-0.8 [c]	-0.79 [b]	0.79 [b]		
Cx43	-0.9 [c]	0.75 [b]	-0.85 [c]	n.s.	-0.59 [a]	-0.65 [a]	0.79 [b]	0.84 [c]	
BNP	0.64 [b]	-0.54 [b]	0.64 [b]	n.s.	0.59 [b]	n.s.	-0.54 [b]	-0.58 [a]	-0.6 [a]

$\hat{A}_{QRS}$, frontal QRS axis; I_{QRS}, QRS amplitude index; T_{QRS}, QRS duration; IS, infarcì size; PWT, posterior (remote) wall thickness; lvEDd, left ventricular end-diastole diameter; FS, percent fractional shortening; SPWT, percent systolic posterior (remote) wall thickening; Cx43, connexin 43; BNP, plasma B-type natriuretic peptide
Correlation was significant at: a, $p<0.05$; b, $p<0.01$; c, $p<0.001$.
